# HIV-1 molecular diversity in Brazil unveiled by 10 years of sampling by the national genotyping network

**DOI:** 10.1038/s41598-021-94542-5

**Published:** 2021-08-04

**Authors:** Tiago Gräf, Gonzalo Bello, Paula Andrade, Ighor Arantes, João Marcos Pereira, Alexandre Bonfim Pinheiro da Silva, Rafael V. Veiga, Diana Mariani, Lídia Theodoro Boullosa, Mônica B. Arruda, José Carlos Couto Fernandez, Ann M. Dennis, David A. Rasmussen, Amilcar Tanuri

**Affiliations:** 1 Fundação Oswaldo Cruz, Instituto Gonçalo Moniz, Rua Waldemar Falcão, 121, Salvador, 40296-710 Brazil; 2 Laboratório de AIDS e Imunologia Molecular, Fundação Oswaldo Cruz, Instituto Oswaldo Cruz, Rio de Janeiro, Brazil; 3 Laboratório de Virologia Molecular, Departamento de Genética-IB, Universidade Federal do Rio de Janeiro, Rio de Janeiro, Brazil; 4 Laboratório de Bioinformática and Evolução Molecular, Departamento de Genética-IB, Universidade Federal do Rio de Janeiro, Rio de Janeiro, Brazil; 5 Center of Data and Knowledge Integration for Health (CIDACS), Fundação Oswaldo Cruz, Instituto Gonçalo Moniz, Salvador, Brazil; 6 Instituto de Ciências da Saúde, Universidade Federal da Bahia, Salvador, Brazil; 7 Division of Infectious Diseases, University of North Carolina, Chapel Hill, NC USA; 8 Department of Entomology and Plant Pathology, North Carolina State University, Raleigh, USA; 9 Bioinformatics Research Center, North Carolina State University, Raleigh, USA

**Keywords:** Viral epidemiology, HIV infections, Phylogenetics

## Abstract

HIV-1 has diversified into several subtypes and recombinant forms that are heterogeneously spread around the world. Understanding the distribution of viral variants and their temporal dynamics can help to design vaccines and monitor changes in viral transmission patterns. Brazil has one of the largest HIV-1 epidemics in the western-world and the molecular features of the virus circulating in the country are still not completely known. Over 50,000 partial HIV-1 genomes sampled between 2008 and 2017 by the Brazilian genotyping network (RENAGENO) were analyzed. Sequences were filtered by quality, duplicate sequences per patient were removed and subtyping was performed with online tools and molecular phylogeny. Association between patients’ demographic data and subtypes were performed by calculating the relative risk in a multinomial analysis and trends in subtype prevalence were tested by Pearson correlation. HIV-1B was found to be the most prevalent subtype throughout the country except in the south, where HIV-1C prevails. An increasing trend in the proportion of HIV-1C and F1 was observed in several regions of the country, while HIV-1B tended to decrease. Men and highly educated individuals were more frequently infected by HIV-1B and non-B variants were more prevalent among women with lower education. Our results suggest that socio-demographic factors partially segregate HIV-1 diversity in Brazil while shaping viral transmission networks. Historical events could explain a preferential circulation of HIV-1B among men who have sex with men (MSM) and non-B variants among heterosexual individuals. In view of an increasing male/female ratio of AIDS cases in Brazil in the last 10–15 years, the decrease of HIV-1B prevalence is surprising and suggests a greater penetrance of non-B subtypes in MSM transmission chains.

## Introduction

One of the key features responsible for HIV-1 becoming pandemic is the virus’s fast mutation rate. This mechanism gives the virus the ability to quickly adapt to the host immune response, acquire resistance to antiretroviral therapy and results in remarkably molecular diversity^1^. Four HIV-1 groups (named M, N, O and P) originated independently from cross-species transmission events between humans and non-human primates around 100 years ago in Central Africa^2^. Among these, group M successfully spread around the world, diversifying into nine subtypes (A–D, F–H, J, and K) and more than 100 circulating recombinant forms (CRFs) (a complete list of HIV-1 CRF is available at: https://www.hiv.lanl.gov/content/sequence/HIV/CRFs/CRFs.html). Currently, the most prevalent HIV-1 variant is subtype C (HIV-1C) (~ 47% of worldwide infections), followed by subtype B (HIV-1B) (~ 12%) and subtype A (~ 10%)^3^. The dominance of HIV-1C is mainly due to its presence in the Southern African countries, where the number of people living with HIV-1 is the greatest, while HIV-1B is the most frequent in middle and high-income countries, such as in the Americas and western Europe.

A complex process of human migrations, motivated by military conflicts and socio-economic factors has shaped HIV-1 diffusion within African countries and out of the continent^4^. Although founder effects can explain much of the heterogeneous distribution of HIV-1 subtypes around the world (i.e. the first to arrive dominates the local epidemic), several inherent viral factors, such as the rate of disease progression, efficiency of transmission and response to antiretroviral therapy (ART), may also influence the successful spread of particular subtypes or CRFs^1^. However, these factors are not well understood yet and a better comprehension of HIV-1 diversity and its implications on viral transmission and disease onset would greatly help pandemic control efforts. Furthermore, regional differences in which HIV-1 lineages circulate impose complex and challenging hurdles to the development of HIV vaccines^5,6^. Thus, an in-depth knowledge of the global viral diversity is instrumental to the prioritization of candidate vaccines with the greatest potential benefits.

Brazil has a population of ~ 210 million people spread over a large geographic area. An estimated 830,000 individuals (0.4%) are currently living with HIV in Brazil, which represents around 40% of all HIV infections in Latin America and the Caribbean combined^7^. Studies on molecular epidemiology have revealed that HIV-1B prevails (~ 67%) in Brazil, followed by HIV-1C (~ 14%) and subtype F1 (HIV-1F1, ~ 10%)^8^. However, large regional heterogeneities are observed in the national HIV-1 diversity^9–13^, which are not well described yet. Besides, no previous study has longitudinally assessed trends in HIV-1 diversity in Brazil and such analysis can provide information about demographic changes in the epidemic or differential fitness of viral lineages. In this study, we present a comprehensive analysis of the HIV-1 molecular diversity in the Brazilian Genotyping Network (RENAGENO) databank that contains information on 46,877 patients in therapeutic failure, pregnant women and children born with HIV that were sampled for routine genotyping service between 2008 and 2017.

## Methods

### Ethical aspects

This work was approved by the Research Ethics Committee of the Instituto Gonçalo Moniz/Fiocruz-BA under Registration No. 15300719.5.0000.0040. A waiver of informed consent was obtained from Research Ethics Committee of the Instituto Gonçalo Moniz/Fiocruz-BA. Data was collected between 2008 and 2017 and analyzed in 2019 and 2020. All methods were performed in accordance with the relevant guidelines and regulations.

### The Brazilian Genotyping Network (RENAGENO) database

Concatenated complete protease (PR) and partial reverse transcriptase (RT) HIV-1 sequences (~ 1100 base pairs) and patients’ metadata were kindly provided by the Department of Diseases of Chronic Condition and Sexually Transmitted Infections, of the Secretariat for Health Surveillance of the Brazilian Ministry of Health. Initially, the database contained 53,413 patients, sampled for routine genotyping services between 2008 and 2017, which is offered as pre-therapy for perinatally infected children and pregnant women, and for virologic failure individuals. Besides HIV-1 nucleotide sequences, the database also contained basic patients' metadata, such as sex, date of birth, sampling location, year of education and color/race. Individuals were identified by an alphanumeric code to preserve confidentiality. Data about the Brazilian HIV-infected population was collected from the DATASUS system, a databank hosted by the Brazilian Ministry of Health (http://www2.datasus.gov.br/DATASUS/index.php?area=0203).

### Data cleaning and sequence quality control

Correspondence between patients’ identifiers and sequence headers were cross-checked and only paired instances were maintained in the analyzed dataset. Next, sequences were submitted to the Los Alamos Quality Control tool (available at: https://www.hiv.lanl.gov). Sequences with more than three frame-shift events or stop-codons were excluded, as well as those identified as hypermutated. Lastly, we removed redundant sampling from patients that underwent genotyping more than once and retained the first generated sequence.

### HIV-1 subtyping

Sequences were initially subtyped using the web-tools REGA Subtyping Tool v3.0 program (as available at: https://www.genomedetective.com)^14^, COMET^15^, and RIP (available at: https://www.hiv.lanl.gov). Subtype assignment was defined when two or more tools agreed. For sequences with conflicting results, the classification was performed by molecular phylogeny. Subtype reference sequences were obtained from Los Alamos HIV Sequence Database (http://www.hiv.lanl.gov/), aligned with the RENAGENO dataset using MAFFT algorithm and visually inspected in Aliview program^16,17^. Maximum likelihood (ML) trees were constructed using IQ-TREE program^18^, as available in the CIPRES Science Gateway platform. Subtype assignment was based on high support (≥ 70 ultrafast bootstrap) clustering with reference sequences. When the positioning of the query sequence in the phylogenetic tree remained inconclusive for subtype classification, the bootscanning method implemented in Simplot v3.5.1 program was applied with window size of 300nt and incremental steps of 20nt^19^. HIV-1B non-pandemic lineage (BCAR), that is, the presence of the rare HIV-1B lineages that were directly seeded from the Caribbean, was identified by combining the RENAGENO HIV-1B sequences with reference datasets of BCAR (n = 200) and BPANDEMIC (n = 300), previously described^20^. Classification was performed based on their placement within either clade on ML phylogenetic trees sequentially inferred as explained elsewhere^21^.

### Statistical analysis

Correlations between HIV-1 subtype occurrence and patients’ socio demographical data was estimated by calculating the relative risk (RR) of an individual being infected by a particular subtype given their demographic variables using a multinomial logistic regression model. HIV-1B was defined as the baseline category and sampling year was combined into three periods. Pearson's correlation test was used to describe temporal trends in HIV-1 subtype prevalence by Brazilian region. Tests were performed between year of sampling and subtype proportion in that year. Aiming to capture changes in subtypes’ transmission reflected in annual trends in subtypes’ proportion, only individuals older than 14 years were analyzed. Due to temporal sampling bias, women and men were analyzed separately.

## Results

### The RENAGENO HIV-infected sampled population

**Figure 1:**
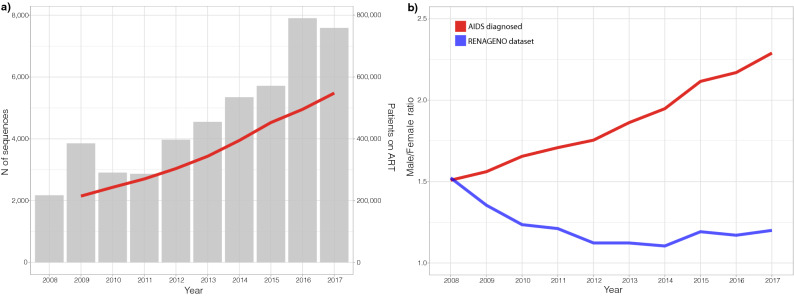
Number of sequences per year in the RENAGENO dataset (gray bars) and number of patients on ART in Brazil (red line), as available from 2009 onwards (**a**). Changes in the male/female ratio through time in reported AIDS cases diagnosed in Brazil and in the RENAGENO dataset (**b**).

After the data cleaning procedure, the dataset included 46,877 HIV-infected individuals sampled between 2008 and 2017. This final dataset of sequences was made available in GenBank under Accession numbers are KEXV01000001 to KEXV01046877. A steep increase in the number of HIV-1 sequences per year can be observed in the RENAGENO dataset (Fig. 1a), closely mirroring the increment of patients on ART in Brazil.

To characterize the current picture of the HIV-1 molecular diversity in Brazil, we compared data from patients sampled in the last triennium (2015–2017) to data of all AIDS diagnosed individuals from the same period, as reported by the Brazilian Ministry of Health (Table 1). Due to the sampling criteria of the genotyping service in Brazil, the RENAGENO dataset is enriched with women and children (≤ 14 years old) (Table 1). While the AIDS epidemic in Brazil has a current (2015–2017 period) male/female ratio of 2.2, the RENAGENO dataset presents a ratio of 1.2. Further, there’s a clear increasing trend in the proportion of men diagnosed with AIDS in Brazil over the last 10 years, while the male/female ratio within the RENAGENO dataset seems to be stable since 2012 (Fig. 1b). Remarkably, 84.8% of all children diagnosed with AIDS in the period of 2015–2017 were present in the RENAGENO dataset. Even enriched with children, the median age of the RENAGENO sampled individuals (38, IQR 18) was slightly higher (*p* < 0.01) than the median age of the AIDS diagnosed individuals (36, IQR 18) (Supplementary Fig. 1), which might reflect the period between ART initialization and ART failure. Despite the high number of missing data, the observed differences in years of education might reflect the high presence of children in the RENAGENO dataset and gender disparity, while differences in color/race might be a consequence of the undersampling of the regions north and northeast.

**Table 1:** Demographic features of the 2015–2017 sampled population in the RENAGENO dataset as compared to the reported AIDS diagnosed individuals in the same period.

	RENAGENO dataset (N = 21,209)	AIDS diagnosed (N = 117,429)
**Sex**		
Male	11,502 (54.3%)	80,612 (68.7%)
Female	9692 (45.7%)	36,792 (31.3%)
NA	15	25
**Age**^**a**^		
≤ 14	1302 (6.1%)	1535 (1.3%)
15–24	1984 (9.4%)	14,932 (12.7%)
25–39	8022 (37.8%)	53,121 (45.2%)
40–69	8929 (42.1%)	41,209 (35.1%)
≥ 60	969 (4.6%)	6632 (5.6%)
NA	3	0
**Years of education**		
zero	871 (7.6%)	1467 (2.7%)
1–3	1372 (11.9%)	4705 (8.6%)
4–7	3975 (34.6%)	13,977 (25.5%)
8–11	3754 (32.7%)	25,035 (45.7%)
12 or more	1514 (13.2%)	9642 (17.6%)
NA	9723	62,603
**Color/race**		
White	6860 (47.8%)	28,921 (42.2%)
Black	1796 (12.5%)	7656 (11.2%)
Asian	112 (0.8%)	316 (0.5%)
Mixed	5543 (38.7%)	31,393 (45.8%)
Amerindian	26 (0.2%)	231 (0.3%)
NA	6872	48,912
**Region**		
North	1664 (7.8%)	13,004 (11.1%)
Northeast	4098 (19.3%)	26,698 (22.7%)
Southeast	9675 (45.6%)	46,264 (39.4%)
South	4308 (20.3%)	23,166 (19.7%)
Central-West	1464 (6.9%)	8297 (7.1%)

*NA* data not available.

^a^When sampled for genotyping or when diagnosed with AIDS.

### The current picture of the HIV-1 molecular diversity in Brazil

**Figure 2:**
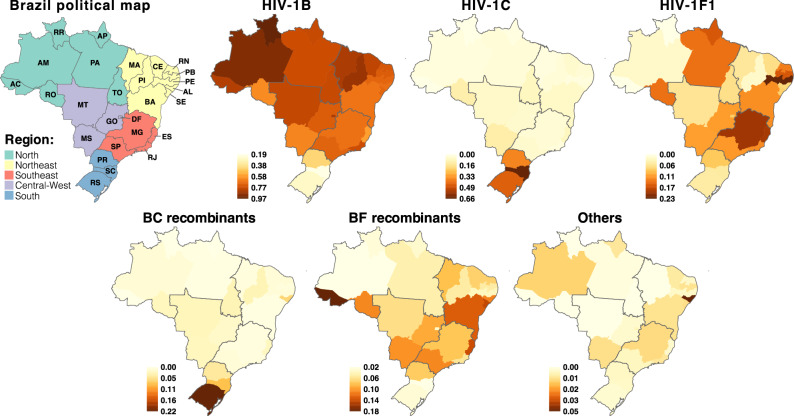
Frequency of HIV-1 subtypes and main recombinant forms in Brazil. Brazilian states are colored according to variant frequency among all sampled sequences in the last triennium of the dataset (2015–2017). The color scale is unique for each viral variant and political division is showed for country regions as in the map on the left. Maps were draw in R (version 4.0.3—https://www.r-project.org) using the libraries tmap, sf and brazilmaps. *AL* Alagoas, *AP* Amapá, *AM* Amazonas, *BA* Bahia, *CE* Ceará, *DF* Distrito Federal, *ES* Espírito Santo, *GO* Goiás, *MA* Maranhão, *MT* Mato Grosso, *MS* Mato Grosso do Sul, *MG* Minas Gerais, *PA* Pará, *PB* Paraíba, *PR* Paraná, *PE* Pernambuco, *PI* Piauí, *RJ* Rio de Janeiro, *RN* Rio Grande do Norte, *RS* Rio Grande do Sul, *RO* Rondônia, *RR* Roraima, *SC* Santa Catarina, *SP* São Paulo, *SE* Sergipe, *TO* Tocantins.

Corroborating previous studies, our findings indicate that three subtypes (HIV-1B, HIV-1C and HIV-1F1) and two groups of recombinant forms (BC and BF) are responsible for 99% of the HIV-1 molecular diversity in Brazil. HIV-1 variants with a frequency smaller than 1% in the whole analyzed period (2008–2017) were classified as “Others”. In the most recent triennium of the RENAGENO dataset (2015–2017) 64.0% of samples were HIV-1B, 13.2% HIV-1C, 10.9% HIV-1F1, 7.3% BF recombinants, 3.9% BC recombinants and 0.7% other forms. This viral diversity is heterogeneously distributed across Brazilian regions and states (Fig. 2). HIV-1B is the dominant form in 25 out of 27 Brazilian states, reaching the highest prevalence in northern states of Amazonas (AM, 91.2%) and Roraima (97.4%); while HIV-1C was dominant in the two southernmost states of Rio Grande do Sul (RS, 44.7%) and Santa Catarina (SC; 66.2%). We also observed high prevalence of HIV-1C in the southern state of Paraná (PR, 36.8%) and medium prevalence (10–15%) in the central-western state of Mato Grosso do Sul (MS) and the northern state of Rondônia (RO). Distinctly from HIV-1C, which is highly concentrated in the south region, HIV-1F1 is widely dispersed across the Brazilian states, accounting for more than 10% of the infections in nearly half of the territory (13/27 states). The states with the highest prevalence of HIV-1F1 are Pernambuco (PE), Minas Gerais (MG) and Espírito Santo (ES), with 23.0%, 19.8% and 19.2% respectively.

HIV-1 recombinant strains were identified up to the CRF level only when clearly clustering with reference sequences or when assigned by two or more online subtyping tools. When none of these criteria were reached, recombinant strains were classified in a generic way based on the subtypes composing its genome as visualized in a bootscanning analysis. Thus, the CRF frequencies presented here are likely to be underestimated and a more thorough analysis of the recombination breakpoints could better classify mosaic strains into the known CRFs. BC recombinant forms were highly prevalent in the RS state (21.9%), where CRF31_BC is responsible for at least 8.9% of the infections. CRF31 is the only BC circulating form characterized in Brazil^22^ so far and, in our analysis, it represented 35.1% of all BC recombinants (data not shown). Despite only being prominent in RS state, CRF31_BC was found in low proportions in 20 out of 27 Brazilian states, in agreement with a previous study^23^. Similar to HIV-1F1, BF recombinants are more dispersed across Brazil and eight states presented frequencies higher than 10%, being particularly notable the high prevalence (18.2%) estimated in the state of Acre. Nine different CRF_BFs were found circulating in Brazil in our analysis. The most frequent were CRF28/29_BF (whose differentiation was not possible in the genomic region analyzed here) and CRF12_BF, representing 15.2% and 6.4% of all BF recombinants. None of the identified CRF_BFs were found to be particularly relevant in the HIV-1 epidemic at the state level.

Among the rare variants (Others), the most frequent were CRF02_AG (N = 42), subtype D (N = 25) and CRF45_cpx (N = 19). The state of Alagoas (AL) showed an interesting epidemic where rare variants represented 4.6% of the HIV-1 diversity (Fig. 2), CRF02_AG (2.6%) being the most frequently found. We also assessed the presence of the non-pandemic HIV-1B lineage in Brazil (also called HIV-1BCAR) in contrast to the most prevalent BPANDEMIC strain that was originally seeded from the USA. The HIV-1BCAR represented 3.4% of all HIV-1B sequences in the RENAGENO dataset in the last triennium, but its presence was much higher in the north region (13.6%), reaching strikingly 29.7% of HIV-1B sequences in Roraima, 25.0% in AM and 12.5% in Acre (AC) states (Supplementary Figure 2).

### Viral diversity and social demographic features

**Figure 3:**
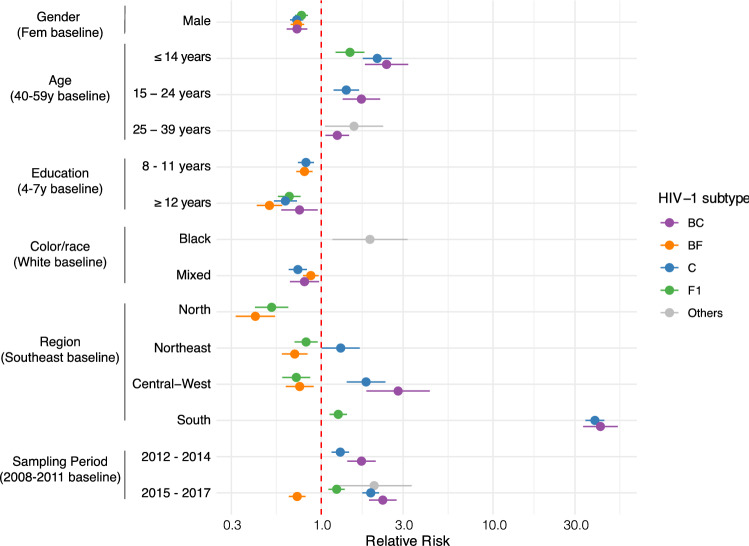
Relative risk of HIV-1 subtypes and main recombinant forms among RENAGENO sampled population. Relative risk and 95% confidence interval were calculated in a multinomial logistic regression model with HIV-1B as the baseline. Baseline level for each social demographic variable are between brackets. Only statistically significant (*p* < 0.05) associations are shown.

We then assessed the potential association of the HIV-1 subtypes with demographic features of the sampled population by calculating the relative risk (RR) in a multinomial logistic regression model with HIV-1B as the baseline category. Being aware of the heterogeneous distribution of HIV-1 subtypes across the Brazilian territory and the changes in the dataset composition across time, the sampling region and year were included in the multinomial analysis. Figure 3 shows only the significant RR and 95% CI. As observed in Fig. 2, there is an evident heterogeneous distribution of subtypes in the country by region, with the highest RR for HIV-1C and BC occurring in south Brazil, followed by Central-West. HIV-1F1 and BF recombinants were less likely to be found in regions North, Northeast and Central-West. Besides that, it is interesting to observe that subtypes C, F1, BC and BF were less likely to be found in males and in individuals with ≥ 12 years of education. HIV-1C and BF recombinants also presented smaller RR to be found in individuals with 8–11 years of education. To control for education inequalities between women and men, we repeated the analysis for the male subset and found the same correlation between HIV-1B and higher education (Supplementary Figure 3).

We also observe a higher RR for HIV-1C, F1 and BC recombinants among children (≤ 14 years old). Infections in this age category are likely to be the result of vertical transmission since sexual activity is absent or very reduced under 15 years. Thus, this association likely reflects the higher RR of these HIV-1 variants in women. Further, young individuals (15–24 years) also presented higher RR to be infected with HIV-1C and BC, while young adults showed higher RR for BC and Other subtypes. Regarding color/race variable, individuals self-declared as mixed color were less likely to be infected with HIV-1C, BC and BF, while black individuals had higher RR to be infected with Other subtypes. The later association might be the result of recent African immigration, since the main subtypes here classified as Others are commonly found in Africa such as HIV-1D, CRF02_AG and CRF45_cpx. Finally, our analysis reveals an increasing RR to be infected with HIV-1C and BC in the 2012–2014 and 2015–2017 periods, and by HIV-1F and Other subtypes in the 2015–2017 period.

### Viral diversity and temporal trends

**Figure 4:**
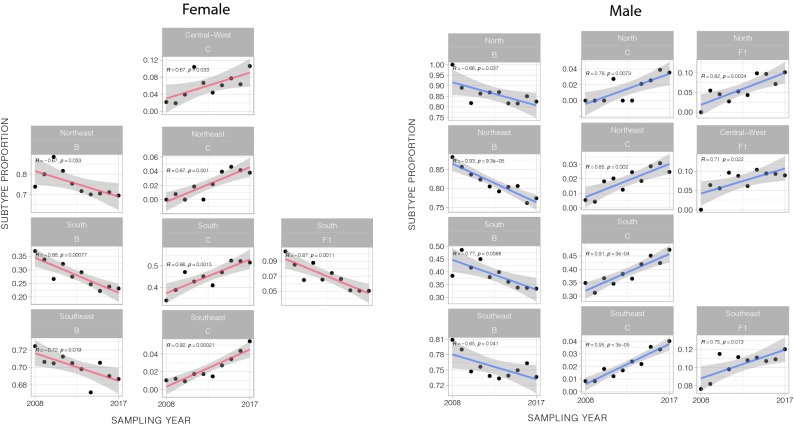
Temporal trends in HIV-1 subtypes proportions in Brazil. Pearson correlation plots between subtype proportion and sampling year (2008–2017) are shown for HIV-1B, C and F1 per Brazilian region. Only statistically significant (*p* < 0.05) correlations are presented. The analysis was performed separately for women (left) and men (right). For the temporal trend of HIV-1 recombinant strains, see Supplementary Figure 4.

Lastly, we explored in more details the variation in the HIV-1 diversity throughout the 10 years of the RENAGENO databank. Due to the observed association between sex and subtypes and changes in the male/female ratio along the sampling period, the correlation analyses between the proportion of HIV-1 variants and sampling year were performed separately in women and men per Brazilian region (Fig. 4). A steady decrease in HIV-1B frequency was observed in both sexes in the northeast, south and southeast regions, and also in men in the north region. The strongest correlation (R [correlation coefficient] = −0.93) was observed among men in the northeast region, where the HIV-1B proportion decreased from 88.2% in 2008, to 77.3% in 2017. In the opposite direction, a rising HIV-1C proportion was observed in women and men in the northeast, south and southeast regions, and also among men in the north region and women in Central-West region. For instance, although still very low in prevalence in the southeast region, HIV-1C showed more than fourfold increase in 10 years, coming from around 1% in both sexes in 2008, to 4.0% in men and 5.4% in women in 2017 (R = 0.95 and 0.92, respectively). Interestingly, significant increase in HIV-1F1 proportion was observed only among men, in all regions but the south and northeast. This subtype increased from proportions below 8% to up to 12.6% with correlation coefficients varying from 0.71 to 0.82. In general, BC recombinants, followed the HIV-1C pattern of increment, despite the even smaller proportions in regions other than the south. BF recombinants were observed to decrease in several regions both in women and men, with exception of the northeast where it increased among men (Supplementary Figure 4).

## Discussion

The Brazilian HIV/AIDS national program is considered exemplary for low- and middle-income countries. Among the several measures to fight AIDS implemented in the early epidemic, the national ART program is one of the most acclaimed worldwide^24^. Since 1996, the Brazilian National Health System universally provides ART, along with viral load and immunological monitoring, as well as viral genotyping for those who fail therapy. In this study we analyzed over 50,000 HIV-1 sequences from Brazilian individuals, representing the most comprehensive HIV molecular epidemiologic study performed to date in the country. Our study brings a clear picture about the distribution of most prevalent subtypes (B, C and F1), recombinants (CRF31_BC, CRF28/29_BF, CRF12_BF) and lineages (HIV-1BCAR and HIVBPANDEMIC) in Brazil. Due to the large sample size, our analyses were able to detect rare HIV-1 subtypes such as subtype D, CRF02_AG and CRF45_cpx that were only sporadically reported in the literature^11,12^.

Despite these strengths, our findings need to be interpreted with caution. First, it is important to highlight that the small HIV-1 genomic region (~ 1100nt) analyzed here very likely underestimates the occurrence of mosaic forms, since any recombination event happening in the other ~ 90% of the HIV genome will not be detected. Second, although the RENAGENO databank broadly represents the Brazilian HIV-infected population, some disparities were observed between this population and new AIDS cases annually reported in Brazil. The main difference was the high proportion of women and children (≤ 14 years) in the RENAGENO dataset (Table 1), which is explained by the genotyping eligible criteria in Brazil. We also observed a lower education level of individuals sampled in the RENAGENO dataset, which could not be explained by sex differences because women are likely to have slightly more years of education than men in Brazil^25^. Instead, lower education level is related to ART poor adherence^26^, which can result in therapeutic failure and consequent inclusion in the RENAGENO dataset. Regional disparities were also observed: the wealthiest southeast region was better sampled in detriment of the poorer north and northeast regions. The later regions present the higher proportion of mixed color individuals, thus explaining their underrepresentation in the RENAGENO dataset.

The HIV-1 diversity is heterogeneously distributed across Brazilian regions and states. HIV-1B is the dominant form in 25 out of 27 Brazilian states, reaching the highest prevalence (> 90%) in northern states, while HIV-1C was dominant (> 44%) in the two southernmost states. BC recombinant forms also reached the highest prevalence in the southernmost state of Rio Grande do Sul (21.9%) and this might be due the role of injecting drug users (IDU) transmission networks, which were shown to be associated to HIV-1C and BC recombinant forms in the 1990’s epidemic in the sate^27^. HIV-1F1 and BF recombinants were widely dispersed across the Brazilian states. The states with the highest prevalence of HIV-1F1 and BF recombinants were Pernambuco (23.0%) and Acre (18.2%), respectively. Details about the remarkable prevalence of HIV-1 subtypes F1 and BF at those Brazilian states should be investigated in future studies. The non-pandemic HIV-1BCAR lineage reached strikingly 25–30% of HIV-1B sequences in Roraima and Amazonas, while the state of Alagoas displayed the highest prevalence of rare variants (4.6%), with CRF02_AG (2.6%) being the most frequently found.

The overall picture of the current HIV molecular diversity in Brazil described here corroborates previous studies that mostly sampled ART naïve individuals and demonstrate that HIV-1B was the dominant form in all Brazilian states, with exception of the two southernmost where HIV-1C prevails^8,12^. The multinomial regression revealed the association of HIV-1B with highly educated men, while non-B variants were more prevalent in women and low-educated individuals. This finding is likely the result of the partial segregation of subtypes in distinct viral transmission networks. The correlation with educational status might reflect socio-economic factors that also shape transmission networks. Although data about sexual orientation was not available, we can infer that HIV-1B preferential circulation among men is driven by men who have sex with men (MSM) transmission networks. Such relation was also observed by several other studies in south Brazil comparing HIV-1C and B epidemics, as reviewed in^28^. The current work suggests that this scenario may be occurring in the whole country. We speculate that the early introduction of HIV-1B in Brazil might have promoted its national-wide dominance among different transmission groups, while the later introduction of HIV-1C and HIV-1F1 variants probably in heterosexual transmission chains, might have had limited penetrance into MSM networks.

Our temporal analysis revealed a dynamic scenario where HIV-1B is steadily diminishing in the most populated regions of the country, while HIV-1C, F1 and BC recombinants are increasing in frequency. Since our sampled population is composed by ART failure patients and HIV diagnosis date was not available in the RENAGENO database, the findings observed here likely reflect transmission events occurring years before sampling and the actual prevalence of these variants might be much higher than currently estimated. Given that non-B subtypes were associated with lower education, one hypothesis would be that the HIV-1 epidemic is growing faster among poorer segments of the population, expanding the transmission networks where these subtypes circulate. However, the Brazilian Ministry of Health data points to the opposite direction, where an increasing proportion of AIDS cases is reported among individuals with more years of education (Sup. Figure 5). A second hypothesis to explain the expansion of HIV-1C, F1 and BC would be an increasing access to MSM transmission networks. The increase in these subtypes among men in several regions of Brazil and the rising male/female ratio of new AIDS diagnosis support this hypothesis. Future studies, using phylogenetics to identify transmission clusters in the RENAGENO dataset could test such hypothesis and characterize the details of viral transmission in social space.

## Conclusions

In summary, our study processed more than 50,000 genomic sequences to reveal the complexity of the HIV-1 molecular diversity in the most populated country of Latin America. We discussed the ongoing changes in the epidemic scenario, highlighting the rise of HIV-1C and F1 over HIV-1B. Such trends are likely promoted by socio-demographic factors that shape viral transmission chains that might also be changing through time. However, we cannot rule out that biological differences between HIV-1 subtypes are shaping viral transmission. For instance, there is accumulating evidence supporting that HIV-1C is relatively attenuated when compared to HIV-1B, which would increase the chances of HIV-1C transmission by slowing down disease progression and increasing the asymptomatic period^29–33^. Important to note, however, that studies on AIDS disease progression are prone to bias due to confounders such as access to medical care, host genetic factors, nutrition status and mode of transmission. Therefore, the hypothesized attenuation of HIV-1C should, ideally, also be tested in the Brazilian infected population before being used as an explanatory variable. Finally, our study highlights the informativeness of using data of national HIV genotyping programs to study changes in genomic diversity and monitor epidemic trends.

## Supplementary Information

Supplementary Figures.
