# Supplementary material for: HIV-1 molecular diversity in Brazil unveiled by 10 years of sampling by the national genotyping network
Source: Sci Rep. 2021 Aug 4;11:15842. doi: 10.1038/s41598-021-94542-5 (PMC8338987; doi:10.1038/s41598-021-94542-5)
Supplement: Supplementary file 1 — Supplementary Figures. [file 41598_2021_94542_MOESM1_ESM.pdf]

Supplementary Material:

HIV-1 molecular diversity in Brazil unveiled by 10 years of sampling by the national genotyping network

Tiago Gräf[1]*; Gonzalo Bello[2]; Paula Andrade[2]; Ighor Arantes[2]; João Pereira [3,4]; Alexandre Bonfim Pinheiro da Silva[3,4]; Rafael V. Veiga[5,6]; Diana Mariani[3]; Lídia Theodoro Boullosa[3]; Mônica B. Arruda[3]; José Carlos Couto Fernandez[2]; Ann M. Dennis[7]; David A. Rasmussen[8,9]; Amilcar Tanuri[3].

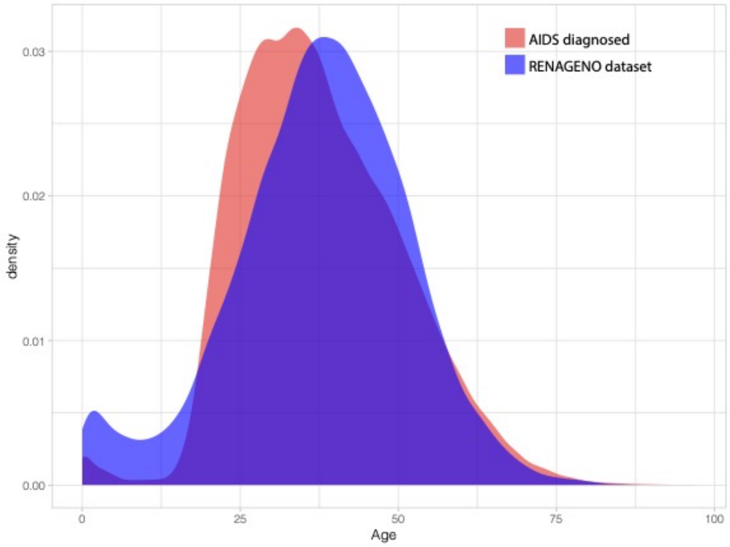

Supplementary figure 1. Age density distribution of all individuals diagnosed with AIDS in Brazil (red) between 2015-2017 and in the RENAGENO dataset (blue) in the same period.

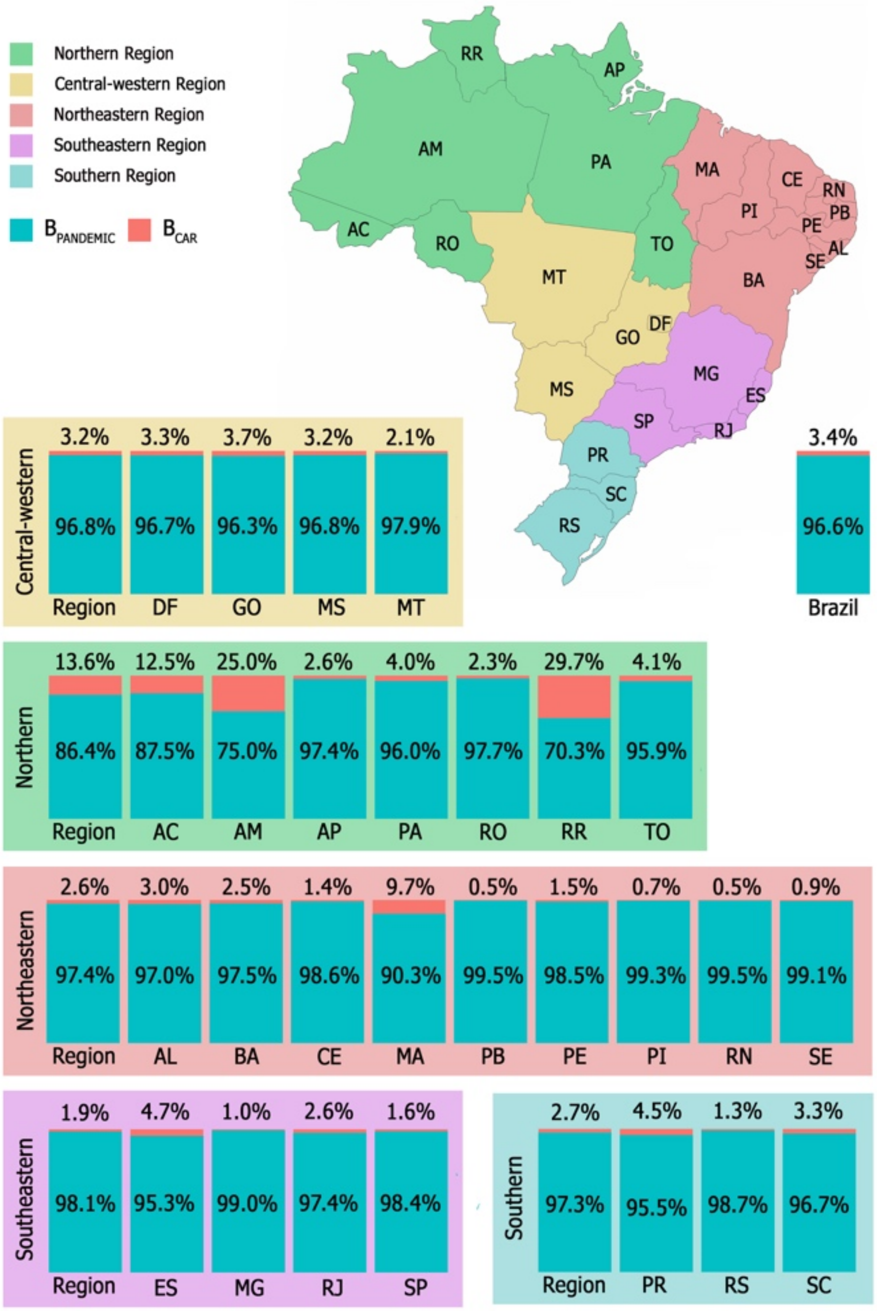

Supplementary figure 2. Proportion of the HIV-1B pandemic ($B_{PANDEMIC}$) and Caribbean (B_{CAR}) lineages per Brazilian sates and regions. Plots and maps were draw in R (version 4.0.3 - https://www.r-project.org) using the libraries ggplot2, tmap, sf and brazilmaps.

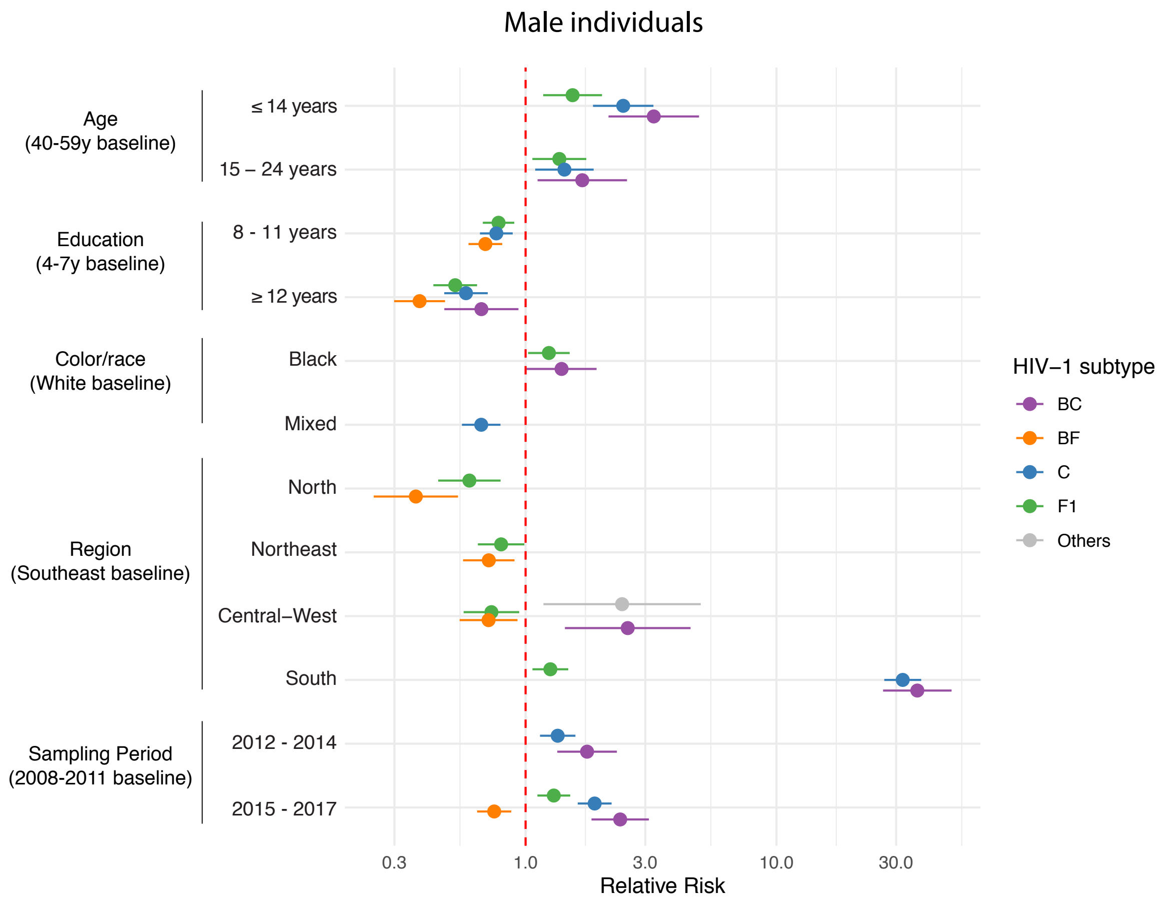

Supplementary figure 3. Relative risk of HIV-1 subtypes and main recombinant forms among RENAGENO male sampled population.

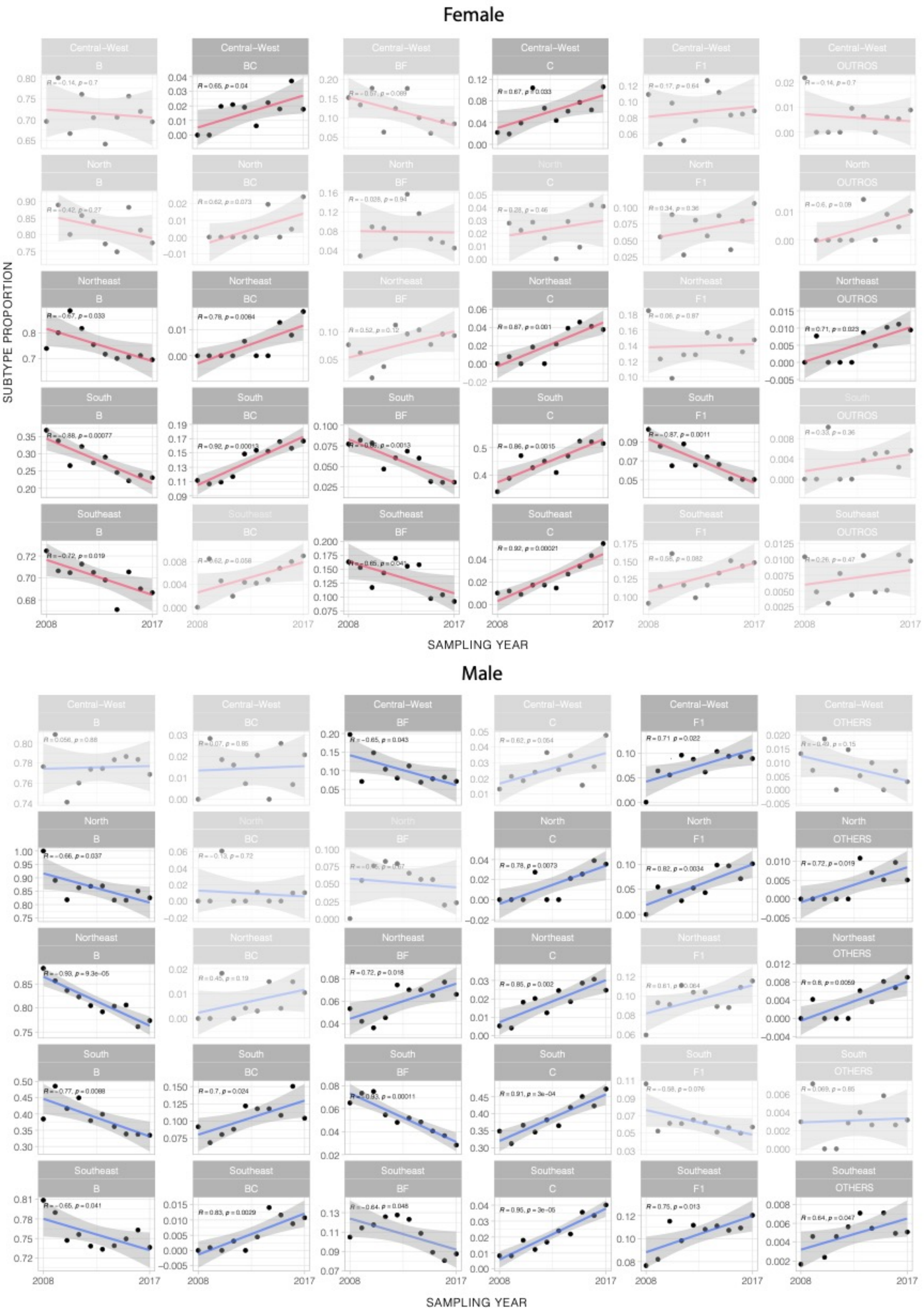

Supplementary figure 4. Pearson correlation between HIV-1 variant proportion and sampling year. Panels are organized by viral variant (columns) and country region (rows). Significant correlations are highlighted and coefficients and *p* values are shown.

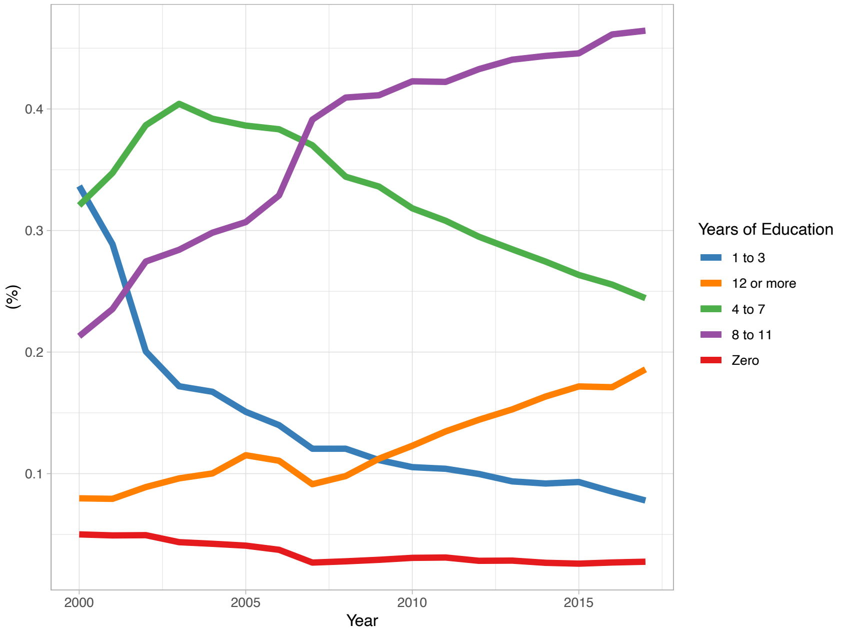

Supplementary figure 5. New AIDS diagnosed individuals in Brazil per education status.
